# Rising tides, cumulative impacts and cascading changes to estuarine ecosystem functions

**DOI:** 10.1038/s41598-017-11058-7

**Published:** 2017-08-31

**Authors:** Theresa A. O’Meara, Jenny R. Hillman, Simon F. Thrush

**Affiliations:** Institute of Marine Science, University of Auckland, Auckland, 1010 New Zealand

## Abstract

In coastal ecosystems, climate change affects multiple environmental factors, yet most predictive models are based on simple cause-and-effect relationships. Multiple stressor scenarios are difficult to predict because they can create a ripple effect through networked ecosystem functions. Estuarine ecosystem function relies on an interconnected network of physical and biological processes. Estuarine habitats play critical roles in service provision and represent global hotspots for organic matter processing, nutrient cycling and primary production. Within these systems, we predicted functional changes in the impacts of land-based stressors, mediated by changing light climate and sediment permeability. Our *in-situ* field experiment manipulated sea level, nutrient supply, and mud content. We used these stressors to determine how interacting environmental stressors influence ecosystem function and compared results with data collected along elevation gradients to substitute space for time. We show non-linear, multi-stressor effects deconstruct networks governing ecosystem function. Sea level rise altered nutrient processing and impacted broader estuarine services ameliorating nutrient and sediment pollution. Our experiment demonstrates how the relationships between nutrient processing and biological/physical controls degrade with environmental stress. Our results emphasise the importance of moving beyond simple physically-forced relationships to assess consequences of climate change in the context of ecosystem interactions and multiple stressors.

## Introduction

As we continue to alter coastal ecosystems, the ability of estuaries to deliver multiple ecosystem services decreases, but our need for the benefits they confer grows. This discrepancy between our reliance on estuaries and their capacity to mitigate change can lead to ecosystem collapse^1–4^. Estuaries play vital roles in regulating nutrient flux. Some estuaries have been shown to reduce nitrogen (N) stocks by over 70% through denitrification alone, mitigating anthropogenic alterations of coastal ecosystems^5–7^. However, estuaries bear the brunt of human induced stress. As the transition between land and sea, estuaries are subject to both terrestrial and marine stressors that can work synergistically to reduce overall functionality and productivity. Climate change (including sea level rise, increased wave action, changes in precipitation patterns, warming, etc.) and intensification of land use promote sediment and nutrient runoff^8–12^. The combination of stressors acting simultaneously constitutes a multiple stressor scenario where predicting the consequences of environmental change on ecosystem function requires examination of the interactions between physical and biological processes that create ecosystem networks. More simplistic single stressor cause-and-effect relationships are likely of limited value because estuarine function is based on complex interactions. Although complexity poses major challenges, we must exploit knowledge of key connections between processes to develop comprehensive models of interaction networks.

Microphytobenthos (MPB) are often the dominant source of primary production on estuarine intertidal flats and play important roles in ecosystem function^13–17^. Shifts from benthic to planktonic production are associated with eutrophication and/or increased turbidity. In estuaries where water column nutrients limit primary production, the MPB regulate ammonia flux across the sediment water interface^17–19^. With their rapid growth, high turnover rate, and high palatability, MPB respond quickly to nutrient pulses and aid in the transfer of nutrients to higher trophic levels for storage, transport and further processing. MPB have other important functional roles in estuarine habitats. The coagulation of surface sediments by mucus produced by MPB reduces nutrient release from buried substrate and regulates turbidity by inhibiting the resuspension of particulates^18^. In healthy estuaries, these regulatory processes are tightly coupled^14, 20^. Healthy ecosystems can cycle excess nutrients through microbial processing, such as denitrification^21–23^. However, in disturbed systems, ecosystem processes may be decoupled. Measuring any single parameter may not provide a complete picture. Therefore, understanding the interactions between ecosystem processes and biological or physical controls provides a more comprehensive descriptor of ecosystem health.

Informed by knowledge of ecosystem processes, we experimentally assessed changes in biological and physical controls of nutrient flux. This involved the deployment of *in-situ* mesocosms (area = 1 m^2^, volume = 180 L) in an experimental design that manipulated sea level (+18 cm), nutrient content (+87 g N m^−2^, 7 g P m^−2^), and mud content (5 mm deposition event) individually and in combination to mimic realistic anthropogenic disturbance on intertidal flats. Experimental results were compared with processes measured along elevation gradients as a method of substituting space for time and assessing the validity of our manipulations. We measured nutrient flux, sediment characteristics, and microphytobenthic production (estimated by benthic chlorophyll-α content) to quantify changes in ecosystem function. We expected a strong correlation between site characteristics and nutrient flux in unaltered (control) experimental plots and transect sites. In contrast, sites altered by increased sea level (SLR), nutrient content, and/or sediment content, were expected to display shifts in the relationships between site characteristics and flux.

## Results

### Benthic production

**Figure 1:**
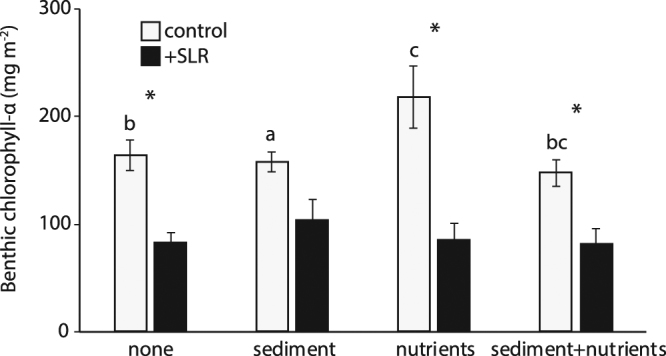
Benthic chlorophyll-α content from all sites. Letters indicate significant differences between controls and *indicate differences between control and SLR treatments (n = 36 per treatment, 288 total).

Benthic production was increased by nutrient additions, decreased by sediment additions, and unchanged when both sediment and nutrients were added (Fig. 1). Sea level had the greatest effect on benthic chlorophyll-α content, and masked any effect of nutrient and sediment additions (Fig. 1). Transect results were consistent with experimental plot data and indicated a decline in MPB production with decreasing elevation/increased inundation (R^2^ = 0.79, p < 0.01, n = 54).

### Modelling ecosystem networks

Generalized linear models were developed using both experimental plot and transect data to determine which interactions were most important in regulating nutrient flux (our proxy for ecosystem function). Models developed of ammonia flux from experimental plots were consistent with transect data and indicated that factors controlling ammonia flux differed between incubations conducted in the light (photosynthetically active, equation 1) and incubations conducted in the dark (photosynthetically inactive, equation 2). Models were developed using all the environmental variables assessed in this study: nutrient fluxes, organic matter content, porosity, O_2_ consumption, and benthic production. However, the most parsimonious models were:

***Light (p < 0.01, R*** ^***2***^ ***= 0.51)***

1 $${\boldsymbol{N}}{{\boldsymbol{H}}}_{{\bf{4}}}^{{\boldsymbol{+}}}={\bf{107.5}}+{\bf{4.9}}({\boldsymbol{P}}{{\boldsymbol{O}}}_{{\bf{4}}}^{{\bf{3}}-})+{\bf{1.5}}({\boldsymbol{chla}})+{\bf{9.4}}({{\boldsymbol{O}}}_{{\bf{2}}})+{\bf{1919.4}}({\boldsymbol{PR}})$$

**Figure 2:**
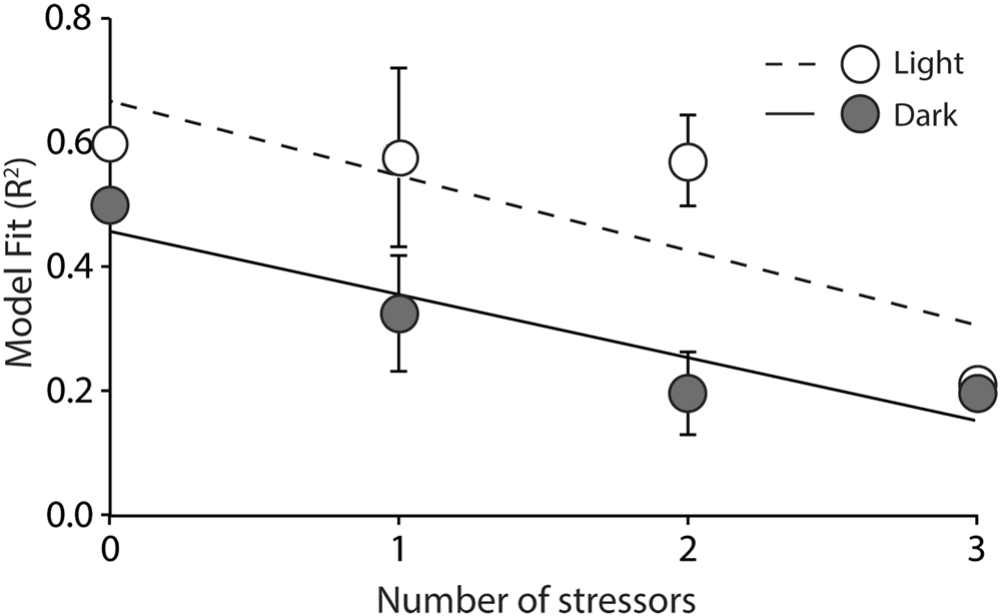
Loss of model strength in response to environmental stress (light: slope = −0.14 R^2^ = 0.62; dark: slope = −0.11, R^2^ = 0.43). Our ability to predict ammonium flux (NH_4_^+^) decreased with added stress, regardless of stressor combination.

***Dark (p*** < ***0.01, R*** ^***2***^ ***= 0.49)***

2 $${\bf{N}}{{\bf{H}}}_{{\bf{4}}}^{+}={\bf{1122.1}}+{\bf{3.7}}({\bf{P}}{{\bf{O}}}_{{\bf{4}}}^{{\bf{3}}-})-{\bf{2348.9}}({\boldsymbol{\rho }})+{\bf{6.0}}({\bf{N}}{{\bf{O}}}_{{\bf{x}}}^{-})+{\bf{38.0}}({{\bf{O}}}_{{\bf{2}}})$$

where PO_4_^3−^ is the flux of soluble reactive phosphorus in ug m^−2^ hr^−1^, chla is the concentration of benthic chlorophyll-α in mg m^−2^, O_2_ is oxygen consumption in mg m^−2^ hr^−1^, PR is photosynthetic rate in g-Carbon (mg chlorophyll-α * hr)^−1^, ρ is porosity (fraction), and NO_x_^−^ is the flux of combined nitrate and nitrite in ug N m^−2^ hr^−1^. The flux of ammonium (NH_4_^+^), under both light and dark conditions, was tightly coupled to soluble reactive phosphorus (SRP) flux and decreased with decreasing elevation. Ammonia flux in light conditions was dominated by primary productivity with chlorophyll-α, O_2_ consumption, and photosynthetic rate as regulatory parameters. Ammonia flux from dark chambers was dominated by respiration, including porosity, which affects organism movement and diffusive flux of NO_x_^−^ produced through microbial processing, and O_2_ consumption. The models for both light and dark NH_4_^+^ were most successful at describing plots with no stressors, and model fit decreased as the number of stressors increased (Fig. 2). The decline in R^2^ occurred regardless of stressor or combination of stressors within each plot.

## Discussion

**Figure 3:**
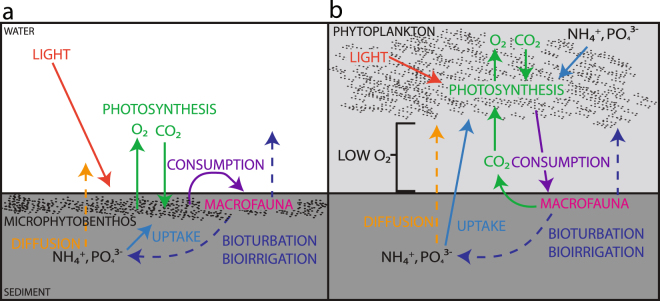
Conceptual network of interactions between nutrients, microphytobenthos and macrofauna inferred from the experiment (**a**) under normal conditions and (**b**) where environmental stress has deemphasised the role of seabed processing. The network and the stability of the system are strongly coupled to microphytobenthic production, which breaks down with stress.

Our experimental plots showed a positive correlation between MPB production and NH_4_^+^ flux, demonstrating the interacting links between MPB, macrofauna, and nutrient flux. MPB regulate NH_4_^+^ flux by capping sediments with mucus, direct uptake, and the provision of food resources to higher trophic levels^24–26^. The tight coupling between SRP and NH_4_^+^ fluxes retained across all experimental treatments is indicative of remineralisation and release across the sediment water interface (Fig. 3a). The observed relationship between porosity in dark chambers and benthic production in light chambers indicates that MPB do play a role in regulating the exchange of nutrients across the sediment water interface in our experimental plots. Benthic grazers also play a role in nutrient flux as they secrete NH_4_^+^ as waste and bioturbate, releasing stored nutrients and oxygenating sediments (Fig. 3a)^27^. Increased food stores attract macrofauna, further increasing bioturbation and the subsequent release of nutrients and greater O_2_ consumption^28–31^. Therefore, an increase in MPB stocks supports benthic grazers and leads to a rise in the release of NH_4_^+^ and SRP from sediments^27, 31, 32^. In our experimental plots, we observed a tight coupling between SRP and O_2_ consumption which is an indicator of bioturbation and bioirrigation. Bioturbating macrofauna consume O_2_ through respiration and their disruption of the anoxic microzone stimulates microbial processing and further O_2_ consumption. The increase in NH_4_^+^ flux observed with increased MPB biomass and greater photosynthetic rate emphasise both the importance of sediment nutrient cycling to primary production in non-eutrophic systems, and the roles of bioturbation and bioirrigation in elevating solute transport rates.

As biogeochemical gradients are altered by benthic grazers, microbial processing of nutrients and organic matter are stimulated. In particular, anammox, coupled nitrification/denitrification, and respiration process nutrients and organic matter released from bioturbation^33–36^. In dark conditions, NO_x_^−^ flux replaced MPB biomass and photosynthetic rate as regulating factors of NH_4_^+^ flux. NH_4_^+^ and NO_x_^−^ are directly released into the water column if the nutrients are not utilized at the sediment water interface. Moreover, nutrients are more readily released from porous sediments, further boosting the flux of both NH_4_^+^ and NO_x_^−^, evidenced by the importance of porosity for dark nutrient flux. In addition to direct release, NO_x_^−^ can be produced through nitrification, which converts NH_4_^+^ to NO_3_^−^, using O_2_ as an electron acceptor. Because benthic O_2_ consumption increases as NO_x_^−^ flux increases in our experiment, this may indicate nitrification occurring within the system. The net result of these changes in flux indicate these systems can shift from a sink of bioavailable nutrients to a source as the interaction network degrades. The models we have developed as a result of our experimental data are informed by knowledge of ecosystem process and illustrate how subtle changes in environmental conditions can shift ecosystems in profound ways (Fig. 3).

The loss of MPB production with SLR in experimental plots and decreased elevation along transects may be linked to a rise in planktonic primary production. In ecosystems reliant on MPB production, a shift from benthic to planktonic production can result in a positive feedback loop. As nutrients are added to the system, primary production shifts to the water column (Fig. 3b)^37, 38^. With the transfer of production from the benthos to the water column, the sediments are shaded and benthic primary production decreases, resulting in increased nutrient release, sediment mobility, turbidity, and eutrophication (Fig. 3b)^1, 13, 39, 40^. Environmental change such as sediment loading and SLR, can thus contribute to the movement of primary production from benthic to planktonic. Suspended sediments increase turbidity and smother benthic algae^8, 11, 25, 27^. Similarly, when intertidal habitat is converted to subtidal, the amount of light available decreases, potentially shifting a higher proportion of production to the water column. This results in the loss of MPB control over nutrient flux across the sediment water interface and can lead to a positive feedback loop fueling eutrophication and a significant shift in the interaction network (Fig. 3b)^41^.

Results show that our ability to predict NH_4_^+^ flux is compromised as the number of stressors increases, which indicates a breakdown in the networked processes governing ecosystem function. Our experiments also indicated that nutrient and sediment additions effects, although very subtle at the low levels used in our experiment, were masked by SLR. The non-linear, non-additive effects of nutrient pollution, sediment addition, and SLR indicate that model predictions of future change will need to weight certain stressors more heavily. Currently, there is no clear way of predicting a tipping point^3, 4^. However, one useful approach is to assess the risk of change to environmental stress in terms of loss of feedbacks and networked interactions^10, 14, 42–44^. Based on our experiment and survey of patterns apparent along gently sloping intertidal to subtidal sandflats, we conclude that SLR is the most important factor in determining an environmental tipping point for primary producers. In this experiment, an 18 cm increase in SLR decreased benthic chlorophyll-α concentrations by ~30%. With SLR estimates ranging from 18–59 cm in New Zealand by 2090^45^, losses in MPB biomass could be higher. As we continue to alter coastal ecosystems, we will observe varied and often unexpected environmental responses. Not only will individual parameters change, but interactions or relationships between these factors will also be altered. We cannot rely on single descriptors to identify tipping points. Instead, we should characterise ecosystem networks to identify at risk communities and potential solutions.

## Methods

Experiments were conducted in Pukapuka Inlet, Mahurangi Harbour, New Zealand (36°29S, 174°43E). Mahurangi Harbour has been monitored extensively since 1994 by the National Institute of Water and Atmosphere (NIWA) for the Auckland Region Council. Technical reports and publications regarding the state of the estuary’s intertidal and subtidal communities can be located on the Auckland Regional Council website (see TR 2009/039, 2009/040, 2013/038, 2016/028 and TP 191 and 209)^46^. The Harbour has a land catchment area of 121 km^2^ and extensive intertidal flats exposed at mean low tide. Experimental plots were established mid-tidally in 2 sites within Pukapuka Inlet in July 2015 (36°29′9.13″S, 174°42′24.59″E and 36°28′36.70″S, 174°42′35.75″E). The experimental plots were established on gently sloping intertidal flats (<63 µm grain size: <15%).

At each site, 3 replicates of each of the following treatments were established: control (no amendments/additions), +nutrients, +sediment, simulated SLR, +nutrients and sediment, +nutrients and SLR, +sediment and SLR, and all three stressors combined (Fig. S1). Once treatments were established, plots settled for 5 months before sampling occurred. Benthic chlorophyll-α content, porosity, and sediment organic matter content were collected during deployment and again in March 2016. Nutrient fluxes were measured in spring (November) 2015 and late summer (March) 2016 to assess flux of NO_x_^−^, SRP, NH_4_^+^, and O_2_ across the sediment water interface. To compare results of *in-situ* SLR manipulations, transects (length = 100 m, sampled every 20 m) were established perpendicular to shore just above the study site and extending to the subtidal zone (Fig. S1). This allowed us to compensate for any artefacts of *in-situ* manipulation of SLR.

### Mimicking environmental change

Estimates of SLR for New Zealand range from 18–59 cm by 2090^45^. To be conservative, we use a simulated SLR of 18 cm. Cylinders (diameter = 1.60 m, height = 0.36 m) were constructed using opaque white HDPE plastic sheets. Cylinders were pushed into sediment until 18 cm of plastic remained above the sediment surface (Fig. S2) to create mesocosms. Due to the sediment grain size and texture, the mesocosm sealed against the sediment, retaining water. Water was exchanged at high tide, but remained subtidal at low tide (Fig. S2). Cylinders were installed to represent 4 different treatments: SLR, SLR + sediments, SLR + nutrients, SLR + sediment + nutrients (Fig. S1 and S2). For treatments with added nutrients, 450 g of controlled release fertiliser (Osmocote Total All Purpose) was inserted 15 cm below the sediment surface using a sediment corer (87 g N m^−2^, 7 g P m^−2^). Nutrient dosage and methodology was adapted from Douglas *et al*. 2016^47^ who performed experiments in similar systems. Sediment additions (adapted from Lohrer *et al*. 2004)^8^ were achieved using slurries of marine mud in seawater. Sediments were collected from the field, sieved to remove shell hash and other large particles, and allowed to sit in fresh water for 2 weeks to neutralise any macrofauna. Sediments were added at low tide and spread evenly across plots to simulate a 5 mm deposition event.

### Sediment Characterization

Sediment samples were collected using a 20 mL syringe to 2.5 cm depth to keep a constant wet volume and dried at 60 °C for 2 days. Porosity was determined using equation (3):

3 $$P=100(\frac{W-D}{V})$$

where *P* is porosity, *V* is volume of wet sediment, *W* is weight of wet sediment, and *D* is weight of dry sediment.

SOM percentages were measured using loss on ignition^48^. Samples were dried at 60 °C for 2 days and combusted at 525 °C for 4 hours. SOM content was determined using equation (4):

4 $$SOM \% =100(\frac{D-M}{D})$$

### Nutrient flux

Concentrations of NH_4_^+^, NO_x_^−^, and SRP were collected twice (November 2015, March 2016) during a midday high tide to maximize photosynthetic capacity. Chambers (diameter = 15 cm, volume = 1 L) were placed over the sediment surface to determine fluxes over approximately 6 hours (Fig. S3). Light and dark chambers were deployed side by side within each plot. Light chambers were constructed of translucent plastic to allow photosynthetic activity to continue. Dark chambers were constructed of black plastic painted white to reduce thermal gradients but stop photosynthetic activity. Flux rates were determined using equation (5).

5 $$Benthic\,Flux=\frac{({C}_{F}-{C}_{I})}{TA}$$

where C_I_ and C_F_ are the initial and final concentrations of an analyte, respectively, T is incubation time (h), and A is the surface area of the core (m^2^)^49^. Initial samples were collected just after deployment and final samples were collected after approximately 6 hours. To account for water column production, ambient samples were collected along with initial samples and water was incubated in light and dark bottles simultaneously with chamber samples. Water samples were filtered using Whatman GF/F filters (pore size of 0.7 µm) and analysed with a Lachat Quick-Chem 8000 automated ion analyser for NO_x_^−^, NH_4_^+^, and PO_4_^3−^.

### Microphytobenthic production

Chlorophyll-α concentrations were used as a proxy for MPB production. Samples were collected just after deployment and again in March 2016. Sediment cores (area = 1.13 cm^2^, depth = 1 cm) were collected in triplicate within each experimental plot and transect site (6 sites per transect). Samples were frozen immediately and processed within 1 month of collection. Chlorophyll-α was extracted from sediments for approximately 18 hours at 0 °C in a solvent mixture of 45:45:10% methanol: acetone: deionised water^21^. After extraction, samples were vigorously mixed and allowed to settle before analysis using a Shimadzu spectrophotometer^50, 51^. Samples were acidified to account for phaeophytin concentrations.

### Statistics

A generalized linear model (GLM) was developed to investigate the individual impacts of each stressor, the combined effects, and determine the relative importance of each. Linear regressions were used to assess trends in benthic production with elevation for transect sites. To determine differences in chlorophyll-*α* content between groups, a Dunn’s Test was conducted. All data were analyzed using R. Errors reported are standard errors.

## Electronic supplementary material

Supplementary Materials

## References

[1] Anderson IC (2014). Impacts of Climate- Related Drivers on the Benthic Nutrient Filter in a Shallow Photic Estuary. Estuaries and Coasts.

[2] Thrush, S. F. *et al*. In *Ecosystem services in New Zealand-conditions and trends* (ed. Dymond, J. R.) 226–237 (Manaai Whenua Press, Lincoln, New Zealand, 2013).

[3] Lenton TM (2011). Early warning of climate tipping points. Nature Climate change.

[4] Kwadijk JCJ (2010). Using adaptation tipping points to prepare for climate change and sea level rise: a case study in the Netherlands. Wiley Interdisciplinary Reviews: Climate Change.

[5] Seitzinger SP (1988). Denitrification in freshwater and coastal marine ecosystems: Ecological and geochemical significance. Limnol. Oceanogr..

[6] Eyre BD, Maher DT, Sanders C (2016). The contribution of denitrification and burial to the nitrogen budgets of three geomorphically distinct A ustralian estuaries: Importance of seagrass habitats. Limnol. Oceanogr..

[7] Nixon SW (1996). The fate of nitrogen and phosphorus at the land-sea margin of the North Atlantic Ocean. Biogeochemistry.

[8] Lohrer AM (2004). Terrestrially derived sediment: response of marine benthic communities to thin terrigenous deposits. Mar Ecol Prog Ser.

[9] Millenium Ecosystem Assessment. Ecosystems and Human Well-being: Wtlands and Water Synthesis (2005).

[10] Moe SJ (2013). Combined and interactive effects of global climate change and toxicants on populations and communities. Environmental Toxicology and Chemistry.

[11] Thrush SF (2004). Muddy Waters: Elevating Sediment Input to Coastal and Estuarine Habitats. Frontiers in Ecology and the Environment.

[12] Titus, J. *et al*. Coastal Sensititvity to Sea-Level Rise: A focus on the Mid-Atlantic Region. *U. S. Climate Change Science Program and the Subcommittee of Global Change Research* U.S. Environmental Protection Agency, 320 (2009).

[13] Underwood GJC, Kromkamp J (1999). Primary Production by Phytoplankton and Microphytobenthos in Estuaries. Adv. Ecol. Res..

[14] Thrush SF, Hewitt JE, Lohrer AM (2012). Interaction networks in coastal soft- sediments highlight the potential for change in ecological resilience. Ecol. Appl..

[15] MacIntyre HL, Geider RJ, Miller DC (1996). Microphytobenthos: The Ecological Role of the “Secret Garden” of Unvegetated, Shallow-Water Marine Habitats. I. Distribution, Abundance and Primary Production. Estuaries.

[16] MacIntyre HL, Cullen JJ (1996). Primary production by suspended and benthic microalgae in a turbid estuary: time-scales of variability in San Antonio Bay, Texas. Mar Ecol Prog Ser.

[17] Barranguet C, Kromkamp J, Peene J (1998). Factors controlling primary production and photosynthetic characteristics of intertidal microphytobenthos. Mar Ecol Prog Ser.

[18] Sundbäck K, Graneli W (1988). Influence of microphytobenthos on the nutrient flux between sediment and water: a laboratory study. Mar Ecol Prog Ser.

[19] Yallop ML, de Winder B, Paterson DM, Stal LJ (1994). Comparative structure, primary production and biogenic stabilization of cohesive and non- cohesive marine sediments inhabited by microphytobenthos. Estuar. Coast. Shelf Sci..

[20] Thrush SF (2003). Habitat change in estuaries: predicting broad-scale responses of intertidal macrofauna to sediment mud content. Mar Ecol Prog Ser.

[21] O’Meara TA, Thompson SP, Piehler MF (2015). Effects of shoreline hardening on nitrogen processing in estuarine marshes of the U.S. mid- Atlantic coast. Wetlands Ecol. Manage..

[22] Koop - Jakobsen K, Giblin AE (2010). The effect of increased nitrate loading on nitrate reduction via denitrification and DNRA in salt marsh sediments.(dissimilatory nitrate reduction to ammonium)(Author abstract)(Report). Limnol. Oceanogr..

[23] Kaplan, W., Valiela, I. & Teal, J. M. Denitrification in a salt marsh ecosystem. *Limnol. Oceanogr*. **24** (1979).

[24] Harris RJ (2015). Biotic interactions influence sediment erodibility on wave-exposed sandflats. Marine ecology progress series.

[25] Serpetti N, Witte UFM, Heath MR (2016). Statistical Modeling of Variability in Sediment-Water Nutrient and Oxygen Fluxes. Frontiers in earth science..

[26] Nicholls, P., Hewitt, J. & Halliday, J. Effects of suspended sediment concentrations on suspension and deposit feeding marine microfauna. *Prepared by NIWA for Auckland Regional Council*. Auckland Regional Council Technical Report 2009/117 (2009).

[27] Lohrer AM, Thrush SF, Gibbs MM (2004). Bioturbators enhance ecosystem function through complex biogeochemical interactions. Nature.

[28] Bolam S, Fernandes T, Huxham M (2002). Diversity, biomass, and ecosystem processes in the marine benthos. Ecol. Monogr..

[29] Savage, C., Thrush, S. F., Lohrer, A. M., Hewitt, J. E. & Lin, S. Ecosystem Services Transcend Boundaries: Estuaries Provide Resource Subsidies and Influence Functional Diversity in Coastal Benthic Communities. *PLoS ONE* **7**(8) (2012). 10.1371/journal.pone.0042708 PMC3411827 22880089

[30] Volkenborn N, Polerecky L, Wethey DS, Woodin SA (2010). Oscillatory porewater bioadvection in marine sediments induced by hydraulic activities of Arenicola marina. Limnol. Oceanogr..

[31] Karlson K, Bonsdorff E, Rosenberg R (2007). The Impact of Benthic Macrofauna for Nutrient Fluxes from Baltic Sea Sediments. Ambio.

[32] Thrush SF, Hewitt JE, Gibbs M, Lundquist C, Norkko A (2006). Functional Role of Large Organisms in Intertidal Communities: Community Effects and Ecosystem Function. Ecosystems.

[33] An S, Joye SB (2001). Enhancement of coupled nitrification-denitrification by benthic photosynthesis in shallow estuarine sediments. Limnology and oceanography..

[34] Joye, S. B. & Anderson, I. C. In *Nitrogen Cycling in Coastal Sediments-Chapter 19* 867–915 (Elsevier Science & Technology, 2008).

[35] Risgaard-Petersen N (2003). Coupled nitrification-denitrification in autotrophic and heterotrophic estuarine sediments: On the influence of benthic microalgae. Limnology and oceanography..

[36] Schlesinger, W. H. In *B*iogeochemis*tr*y an *analysis of global change* (ed. Bernhardt, E. S.) (Waltham, MA: Academic Press c2013, Waltham, MA, 2013).

[37] McGlathery, K. J., Sundback, K. & Anderson, I. C. In *The Influence of Primary Producers on Estuarine Nutrient* Cycling (eds Nielsen, S. L., Banta, G. M. & Pedersen, M. F.) 231-261 (Kluwer Academic, Norwell, 2004).

[38] Genkai-Kato M, Vadeboncoeur Y, Liboriussen L, Jeppesen E (2012). Benthic–planktonic coupling, regime shifts, and whole-lake primary production in shallow lakes. Ecology.

[39] Underwood GJC (2002). Adaptations of tropical marine microphytobenthic assemblages along a gradient of light and nutrient availability in Suva Lagoon, Fiji. Eur. J. Phycol..

[40] Hautier, Y., Niklaus, P. A. & Hector, A. Competition for light causes plant biodiversity loss after eutrophication. *Science*, 636-638 (2009). 10.1126/science.1169640 19407202

[41] Jäger CG, Diehl S, Schmidt GM (2008). Influence of water-column depth and mixing on phytoplankton biomass, community composition, and nutrients. Limnology and oceanography..

[42] Halpern BS (2008). A global map of human impact on marine ecosystems.(REPORTS)(Author abstract)(Report). Science.

[43] Mooney, H. A. In *Biodiversity and Ecosystem Function* (Berlin, Heidelberg: Springer Berlin Heidelberg 1993, Berlin, Heidelberg, 1993).

[44] Sundbäck K, Alsterberg C, Larson F (2010). Effects of multiple stressors on marine shallow- water sediments: Response of microalgae and meiofauna to nutrient– toxicant exposure. J. Exp. Mar. Biol. Ecol..

[45] Parry, M. L. Climate change 2007: impacts, adaptation and vulnerability: contribution of Working Group II to the fourth assessment report of the Intergovernmental Panel on Climate Change. *Intergovernmental Panel on Climate Change: Working Group II* (2007).

[46] Auckland Regional Council. Technical Publications and Research. (16/03/2017) http://www.aucklandcouncil.govt.nz/EN/planspoliciesprojects/reports/technicalpublications/Pages/home.aspx (2017).

[47] Douglas EJ, Pilditch CA, Hines LV, Kraan C, Thrush SF (2016). *In situ* soft sediment nutrient enrichment: A unified approach to eutrophication field experiments. Mar. Pollut. Bull..

[48] Ball DF (1964). Loss‐on‐ ignition as an estimate of organic matter and organic carbon in non-calcereous soils. J. Soil Sci..

[49] Miller-Way T, Twilley RR (1996). A comparison of batch and continuous flow methodologies for determining benthic fluxes. Mar Ecol Prog Ser.

[50] Welschmeyer N, Goericke R, Strom S, Peterson W (1991). Phytoplankton growth and herbivory in the subarctic Pacific: A chemotaxonomic analysis. Limnol. Oceanogr..

[51] Pinckney J, Zingmark RG (1993). Biomass and production of benthic microalgal communities in estuarine habitats. Estuaries.

