# Supplementary material for: Rising tides, cumulative impacts and cascading changes to estuarine ecosystem functions
Source: Sci Rep. 2017 Aug 31;7:10218. doi: 10.1038/s41598-017-11058-7 (PMC5578963; doi:10.1038/s41598-017-11058-7)
Supplement: Supplementary file 1 — Supplementary Materials [file 41598_2017_11058_MOESM1_ESM.pdf]

Supplementary Materials

Rising tides, cumulative impacts and cascading changes to estuarine ecosystem functions

Theresa A. O'Meara*, Jenny R. Hillman, and Simon F. Thrush
Institute of Marine Science, University of Auckland, Auckland 1010, New Zealand
*tome518@aucklanduni.ac.nz

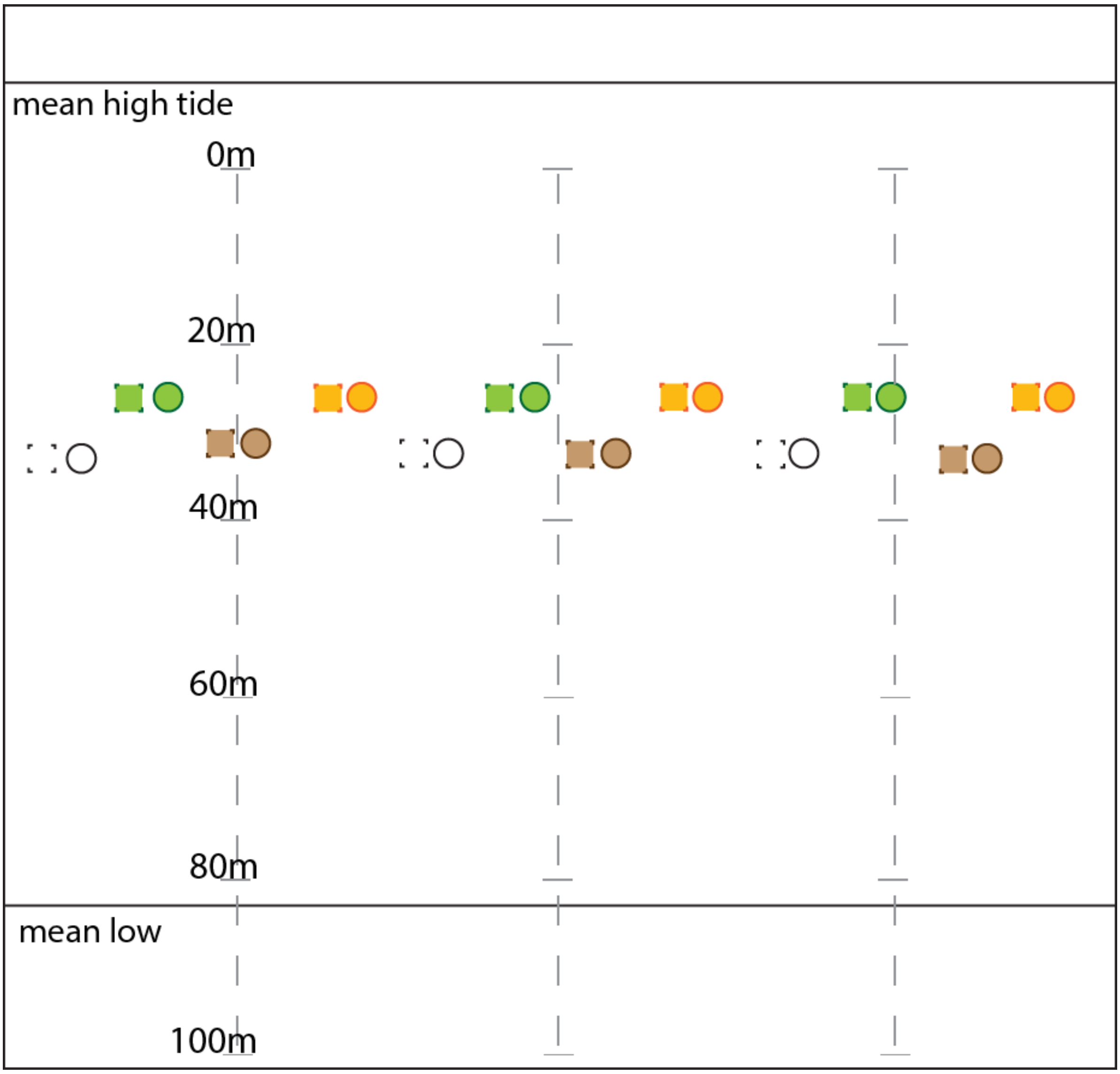

Figure S1. Site layout including experimental plots and transects. (☐) represent mesocosm free plots, (○) represent sites with a mesocosm, and (- - -) indicates transect locations. White plots represent sites without amendments, green plots indicate nutrient addition, brown plots represent plots with sediment added, and yellow indicates plots with both sediment and nutrients added.

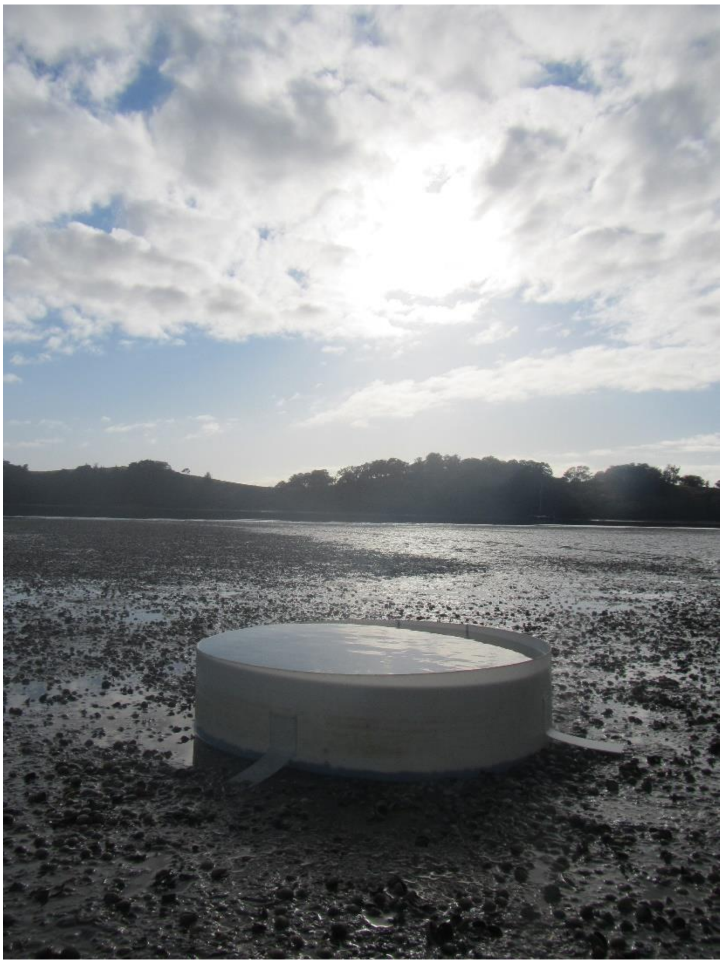

Figure S2. Deployed mesocosm. Note that the mesocosm retains water at low tide, but can exchanges with overlying water at high tide. Photo credit: Jenny Hillman

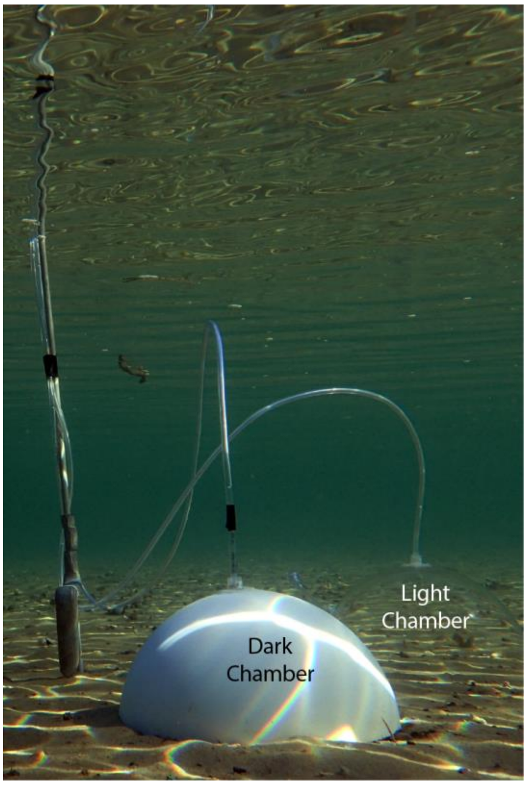

Figure S3. Nutrient chambers. The dark chamber (black painted white) is on the left and the light chamber (clear) is on the right. Photo credit: Jenny Hillman
